# Cost-Effectiveness Evaluation of Etoricoxib versus Celecoxib and Nonselective NSAIDs in the Treatment of Ankylosing Spondylitis in Norway

**DOI:** 10.1155/2011/160326

**Published:** 2011-06-24

**Authors:** Jeroen P. Jansen, Stephanie D. Taylor

**Affiliations:** ^1^Mapi Values, Boston, MA 02114, USA; ^2^Global Health Outcomes, Outcome Research, Merck & Co., Inc., One Merck Drive, P. O. Box 100, WS2E-85, Whitehouse Station, NJ 08889, USA

## Abstract

*Objectives*. To evaluate the cost-effectiveness of etoricoxib (90 mg) relative to celecoxib (200/400 mg), and the nonselective NSAIDs naproxen (1000 mg) and diclofenac (150 mg) in the initial treatment of ankylosing spondylitis in Norway. *Methods*. A previously developed Markov state-transition model was used to estimate costs and benefits associated with initiating treatment with the different competing NSAIDs. Efficacy, gastrointestinal and cardiovascular safety, and resource use data were obtained from the literature. Data from different studies were synthesized and translated into direct costs and quality adjusted life years by means of a Bayesian comprehensive decision modeling approach. *Results*. Over a 30-year time horizon, etoricoxib is associated with about 0.4 more quality adjusted life years than the other interventions. At 1 year, naproxen is the most cost-saving strategy. However, etoricoxib is cost and quality adjusted life year saving relative to celecoxib, as well as diclofenac and naproxen after 5 years of follow-up. For a willingness-to-pay ceiling ratio of 200,000 Norwegian krones per quality adjusted life year, there is a >95% probability that etoricoxib is the most-cost-effective treatment when a time horizon of 5 or more years is considered. *Conclusions*. Etoricoxib is the most cost-effective NSAID for initiating treatment of ankylosing spondylitis in Norway.

## 1. Introduction

Ankylosing spondylitis (AS) is a chronic inflammatory rheumatic disease that affects the axial skeleton, causing characteristics inflammatory back pain, which can lead to structural and functional impairments. Asymmetric peripheral arthritis is present in about 20–40% of patients with AS [1]. In Europe, estimates of prevalence of ankylosing spondylitis vary by tenfold from 0.08% to 0.86% [2–4]. The direct costs of AS are substantial. In Europe, the annual total direct costs per patient have been estimated at €1,800 to €2,800 [5]. The introduction of antitumour necrosis factor alpha (anti-TNF*α*) agents has increased the direct cost of AS [6]. 

The first-line treatment of AS is nonsteroidal anti-inflammatory drugs (NSAIDs) [7–9]. Patients with severe disease refractory to NSAIDs are eligible for anti-TNF*α* agents [10]. The nonselective NSAIDs (nsNSAIDs) have been associated with an increased risk of gastrointestinal (GI) adverse events such as ulcers and GI bleeding because of their inhibition of the gastroprotective COX-1 isoform. COX-2 selective inhibitors were developed with reduced GI toxicity when compared with nonselective NSAIDs. Etoricoxib and celecoxib have been demonstrated to have a superior upper gastrointestinal (GI) safety profile [11–14]. The MEDAL (Multinational Etoricoxib and Diclofenac Arthritis Long Term) program demonstrated the risk of cardiovascular (CV) events with etoricoxib (60 mg/d and 90 mg/d) comparable to diclofenac (75 mg bid and 50 mg tid) [15]. On the efficacy side, etoricoxib has been shown, at doses of 90 mg and 120 mg, to be superior compared to naproxen 1000 mg in the treatment of AS [16]. Celecoxib (200 mg and 400 mg) showed comparable efficacy to diclofenac (150 mg) [17]. 

Given the economic burden of AS, a cost-effectiveness analysis of interventions for AS is warranted. The objective of this study was to evaluate the cost-effectiveness of etoricoxib (90 mg) compared to celecoxib (200 and 400 mg), diclofenac (150 mg), and naproxen (1000 mg) in the treatment of patients with AS in Norway. Analyses were performed from the health care perspective.

## 2. Methods

In the present economic evaluation, a comprehensive decision Bayesian modelling approach was used which integrates evidence synthesis and parameter estimation for efficacy and safety with cost-effectiveness modeling in a single unified framework [18].

### 2.1. Markov Model Description

**Figure 1:**
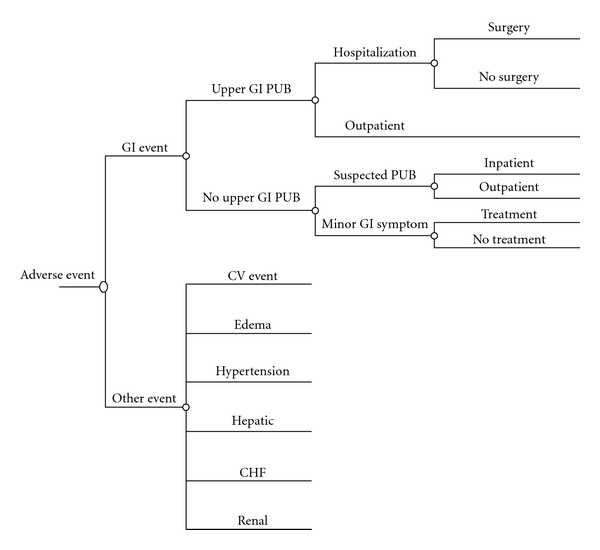
Tree structure reflecting events resulting in costs and potential changes in treatment (i.e., transitions between health states of the Markov model).

A previously published Markov-state transition model was used to estimate the cost-effectiveness of etoricoxib versus celecoxib and nsNSAIDs in the treatment of AS patients requiring daily NSAID treatment [19]. The model consisted of eight health states reflecting treatment received: (1) “initial NSAID” (etoricoxib, celecoxib, or nsNSAIDs, depending on intervention arm of the model), (2) “initial NSAID with proton-pump inhibitor (PPI),” (3) alternative nsNSAIDs with PPI, (4) alternative nsNSAID with PPI and aspirin, (5) alternative nsNSAID, (6) anti-TNF*α* treatment, (7) discontinued anti-TNF*α* treatment, and (8) death. All patients start in health state 1. Transitions from state to state were determined by lack of treatment efficacy, and the different types of events as presented in Table 1. Figure 1 presents the different types of cost generating GI, CV, and other events relevant to each Markov cycle. 

For each health state, utilities were assigned based on the Bath Ankylosing Spondylitis Functional Index (BASFI) and the Bath Ankylosing Spondylitis Disease Activity Index (BASDAI) [20]. Over time, BASFI will worsen thereby decreasing utilities. Disutilities were assigned based on occurrence of adverse events. Drug acquisition costs, and cost due to adverse events were taken into account. 

The model was developed with a cycle length of 1 year. The model followed individuals for a maximum of 30 cycles (30 years) as by this time the majority of individuals had reached the absorbing state (i.e., death). 

### 2.2. Source Data

#### 2.2.1. Efficacy: BASFI, BASDAI, and Discontinuation due to Lack of Efficacy

The efficacy of etoricoxib, celecoxib, diclofenac, or naproxen in AS regarding BASFI, BASDAI, and discontinuation was obtained from a previously performed systematic review and Bayesian mixed treatment comparison (MTC) of randomized controlled trials using noninformative prior distributions [19, 21, 22]. In Table 2 the individual study results are presented. In Table 3, the results of the MTC as used in the cost-effectiveness analysis are presented. 

For the model analysis, the expected change from baseline (CFB) estimates for BASFI and BASDAI by treatment were subtracted from background BASFI and BASDAI values which develop over time. Over time, an increase in BASFI of 0.5 (scale 0–100) per annum was assumed [6, 20]. It was assumed that background BASDAI scores remained stable over time [16, 23, 24]. For patients who continue responding to treatment, it is assumed that their treatment effect regarding BASFI and BASDAI (i.e., the CFB scores) remain constant over time. Patients who switched to another nsNSAIDs were assumed to have the average treatment effect of diclofenac and naproxen as obtained from the MTC. For patients that switched to anti-TNF*α*, a treatment effect of 23 points and 19 points was used for BASFI and BASDAI, respectively. 

It was assumed that 10% withdraw from anti-TNF*α* each year [20, 25]. For patients who withdraw from anti-TNF*α* treatment BASDAI, and BASFI measurements revert back to baseline values as reported by Ara et al. [20]. 

#### 2.2.2. Safety

An overview of all event-related parameters is presented in Table 3. Incidence rates of an upper GI perforation, ulcer, or bleeding (PUB) for etoricoxib, celecoxib, and the nsNSAIDs were estimated with an indirect comparison of the relative incidence rates versus placebo as reported by Ramey et al. (OA, RA, and AS patients) and Silverstein et al. (OA and RA patients) [11, 14, 19]. Incidence rates for suspected PUBs were calculated by subtracting the PUBS from all-investigator-reported PUBs by Ramey et al. [11]. Rates of minor GI symptoms were based on discontinuations due to clinical GI events in the MEDAL programme [28]. Except for PUBs, the rates for other upper GI events with etoricoxib were also used for the celecoxib arm of the model. The probabilities of treatment of GI events were based on Moore et al. [29]. 

The incidence of a thrombotic CV event with etoricoxib, diclofenac, and naproxen were obtained by performing an indirect comparison of the results from the MEDAL programme by Cannon et al. and the relative incidence rate of etoricoxib versus naproxen from a meta-analysis of thrombotic CV events in 12 phase II-IV clinical trials [15, 19, 30]. Rates for etoricoxib were also used for the celecoxib arm of the model. The occurrence of edema, hypertension, coronary heart failure, hepatic adverse events, and renal events were obtained from the MEDAL programme as well [15]. For anti-TNF*α* treatment, and treatment after anti-TNF*α*, no adverse events were taken into consideration. 

The adverse event rate for second-line nsNSAID therapy was assumed to be equal to the average of those obtained for diclofenac and naproxen, with the exception of the incidence of an upper GI event in a patient receiving nsNSAID plus PPI therapy, which was assumed to be reduced by 40% [29]. The GI and CV risk for a patient who switched to nsNSAID plus aspirin and PPI was assumed to be comparable to that for nsNSAID alone.

#### 2.2.3. Mortality

The case-fatality of a UGI PUB or LGI Bleed was 3.6% [29, 31]. A 13% case-fatality for a CV event was used for etoricoxib and celecoxib, and 12.8% case-fatality for nsNSAIDs [15]. For patients not experiencing GI or CV adverse events, a Norwegian annual age-dependent mortality was used, as obtained from the life tables for Norway [32]. For the evaluation, an AS population with an average age of 45 was assumed (base-case scenario) [16].

#### 2.2.4. Utilities

Utilities reflect the preference for a certain health state and are measured on 0-1 scale. A value of 1 reflects perfect health and 0 represents death. By summarizing the utility value over time, quality adjusted life years (QALYs) are created. Life years were transformed into QALYs using a relation between utility (EQ-5d) and BASFI and BASDAI as derived by Ara et al. [20]: Utility = 0.923 − 0.004*BASFI − 0.004*BASDAI. Utility loss associated with adverse events was obtained from the literature [29, 33–36].

#### 2.2.5. Costs

Annual drug acquisition costs were calculated based on the most commonly prescribed drug within a drug class and obtained from the Norwegian Medicines Agency (NoMA September 2007). For anti-TNF treatment, annual costs of etanercept were used. For each type of GI event, numbers of units of health care resource use were assigned and respective unit costs applied to all healthcare resources to calculate the cost per event. The key cost items for GI events included costs of treatment (drugs and dispensing), GP consultations, investigations, inpatient days, and surgery. Costs of thrombotic CV events were weighted according to rates in the MEDAL study. All costs of adverse events were limited to the first year. Cost for the other adverse events (i.e., edema, hypertension, hepatic, and renal) were not taken into consideration. Drug costs related to adverse events were obtained from NoMA (September 2007); costs related to GP visits were obtained from the Norwegian Medical Association (July 2007), and inpatient costs related to events were obtained from the DRG price list [37]. All costs were expressed in 2007 Norwegian kroner (NOK).

### 2.3. Estimating Model Outcomes

Given the Markov state-transition model structure, the source data were combined and translated into the following outcomes: quality adjusted life years, drug acquisition costs, costs of adverse events, total costs, and net monetary benefit (NMB) calculated as QALYs multiplied with a willingness-to pay ratio (WTP) minus costs. WTP is the amount that decision makers are willing-to pay per additional QALY gained. Effects and costs were all discounted at 4% in the base-case scenario.

Since the model was fully probabilistic, outcomes were estimated with MCMC simulation using WinBUGS v 1.4. For each iteration of the model, new parameter values were sampled from the estimated (posterior) or defined distributions for efficacy, safety, and costs (see Table 3). The model was evaluated by averaging output values over many iterations (i.e., 10.000), allowing uncertainty in model parameters to be accounted for. For each iteration, the QALYs and cost accrued for each cycle were calculated for each of the treatments according to Sonnenberg and Beck [38]. At the end of each iteration, the cumulative QALYs and costs over the cycles were obtained by summing the results over all cycles. Next, the incremental cost, incremental QALYs, and incremental cost-effectiveness ratio (ICER = incremental costs/incremental QALYs) of etoricoxib versus the other interventions were evaluated. The probability of cost-effectiveness was expressed with cost-effectiveness acceptability curves, calculated as the number of iterations out of the total number of iterations for which the NMB was greatest for a given treatment out of all 4 treatments. Furthermore, analysis were performed to identify the impact of uncertainty in the source data on the uncertainty in the QALYs, costs, and NMB estimates. 

In the base-case scenario, etoricoxib (90 mg) was compared with celecoxib (200 mg & 400 mg), diclofenac (150 mg), and naproxen (1000 mg). In alternative analyses, the following scenarios were evaluated: (1) celecoxib 200 mg was used instead of celecoxib 200 mg/400 mg, (2) only GI events, (3) only CV events, (4) no adverse events, (5) no discounting on costs and effects, (6) 8% discounting on costs and effects, (7) stable BASFI over time, and (8) assuming an age of 20 years, and (9) anti-TNF*α* costs excluded. For each scenario uncertainty in input parameters was taken into consideration, as outlined above.

## 3. Results

### 3.1. Base-Case Scenario

In Table 4, the results of the base-case scenario are presented for 1 year, 5 years, and 30 years of follow-up. There was more than 98% probability that etoricoxib resulted in higher expected QALYs than the other interventions of interest. 

 Drug costs are expected to be the highest with celecoxib (200 & 400 mg) followed by etoricoxib (90 mg). The nsNSAIDs result in the lowest drug costs. After 5 years, however, the lowest drug costs can be expected for the patients for whom treatment was initiated with etoricoxib due to the higher probability of staying on initial therapy and not switching to the far more expensive anti-TNF*α* treatment. After 30 years, the difference favoring etoricoxib was even greater. 

Relative to a patient starting with nsNSAIDs, the costs due to GI events were lower for a patient starting with etoricoxib or celecoxib as a result of a reduced risk of treatment-requiring GI events. After 30 years, the GI-related costs with etoricoxib were higher than with celecoxib because this latter group of patients switched quicker to anti-TNF*α*, which is not associated with GI events. Until 5 years, costs related to thrombotic CV events were similar with etoricoxib, celecoxib, and diclofenac, and slightly higher than with naproxen. For the same reason as the GI-related costs, the CV-related costs with etoricoxib were higher than with celecoxib and diclofenac at 30 years of followup. Overall, naproxen resulted in the lowest cost at 1 year; however, at 5 years and beyond, etoricoxib resulted in the lowest direct costs of the four alternatives; at 5 year, there is a >96% probability that the lowest costs are obtained with etoricoxib. This increased to >99% at 30 years. 

**Figure 2:**
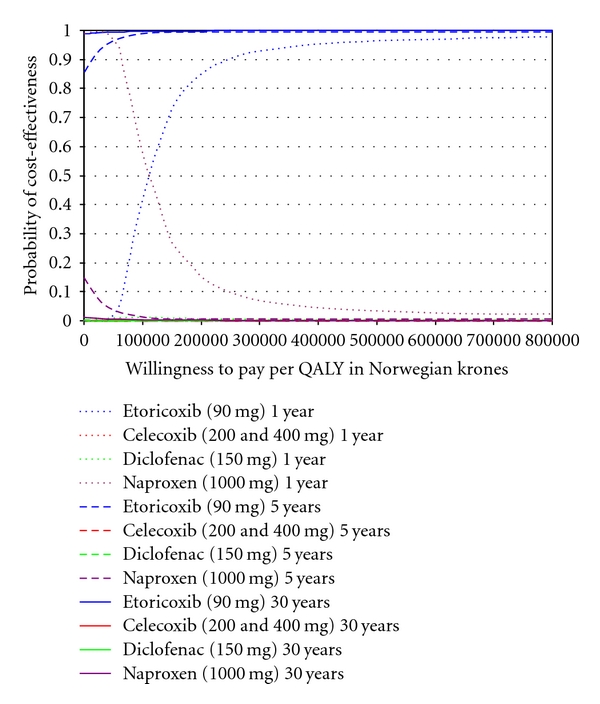
Cost-effectiveness acceptability curves reflecting the probability of cost-effectiveness for etoricoxib, celecoxib (200 & 400 mg), diclofenac, and naproxen at a followup of 1 year, 5 years, and 30 years (base-case scenario).

**Figure 3:**
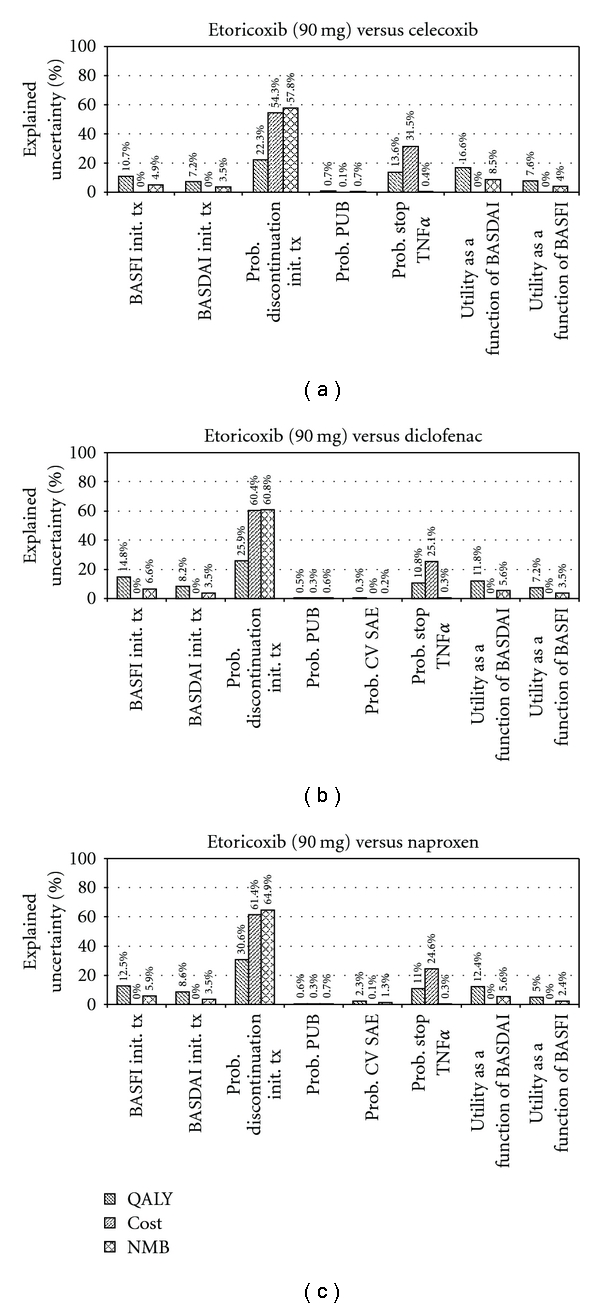
Proportion of explained uncertainty in model outcomes (incremental QALYs, costs, and net-monetary benefit at WTP of 400,000 NOK) by the most relevant variables for the comparison of etoricoxib (90 mg) versus celecoxib, diclofenac, and naproxen (base-case scenario).

In Table 5, the difference in costs and QALYs of etoricoxib relative to the other interventions is presented. Given the more favourable outcomes regarding costs and QALYs with etoricoxib after 5 years of followup, etoricoxib is considered an economically dominant intervention. At 1 year, etoricoxib is economically dominant over celecoxib. The incremental cost-utility ratio (ICER) of etoricoxib relative to diclofenac and naproxen at 1 year was 59,221 NOK and 107,256 NOK, respectively. In Figure 2, the probability of cost-effectiveness for the different interventions at different willingness-to-pay (WTP) ratios are presented. For a WTP 200,000 NOK per QALY there is an 85% probability that etoricoxib is the most cost-effective intervention at 1 year. This increased to more than 96% for WTP of 500,000 NOK and higher. At 5 and 30 years, there is a more than 99% probability that etoricoxib is the most cost-effective intervention.  Figure 3 provides an overview which of the parameters have the greatest impact on (uncertainty) in model outcomes.

#### 3.1.1. Alternative Scenarios

When celecoxib 200 mg was used as a comparator instead of celecoxib 200 mg & 400 mg combined, etoricoxib was no longer economically dominant at 1 year. Etoricoxib is 1,322 NOK more expensive. Given the QALY gain of 0.07, this translates into a cost per QALY of 17,882 NOK, a cost-effective result.

Scenarios where (1) only GI events were included as adverse events, (2) only CV events were included as adverse events, (3) no adverse events were included, (4) no discounting was applied, (5) 8% discounting was applied, (6) BASFI was assumed to be stable over time, and (7) assuming an average age of 20 years provided comparable cost-effectiveness results as the base-case analysis. Only for the scenario where anti-TNF*α* costs were set to zero, etoricoxib was no longer dominant. However, etoricoxib can still be considered cost-effective, independent of the time horizon (see Table 6).

## 4. Discussion

The economic evaluation demonstrated that etoricoxib (90 mg) is an economically superior treatment of AS to celecoxib (200 & 400 mg), diclofenac (150 mg), and naproxen (1000 mg) for both QALY gains and cost savings for a time horizon longer than 5 years. For a 1-year time horizon, etoricoxib is associated with greater costs than diclofenac (150 mg) and naproxen (1000 mg), but can still be considered cost effective.

 In addition to drug acquisition costs for the NSAIDs, also costs for anti-TNF*α* treatment after failure on NSAIDs were taken into consideration. Given the model structure we opted for, the average duration on any NSAID was estimated to be 11.2 years (95% CrI 9.0–13.2) for the patients starting with etoricoxib, 7.8 (6.8–9.0) years with celecoxib, 8.3 (6.8–10.1) years with diclofenac, and 8.4 (6.9–10.2) years with naproxen. These differences explain the savings regarding drug acquisition observed when initiating treatment with etoricoxib over the other treatment strategies observed at the 5,- and 30-year time horizon. However, when the anti-TNF*α* acquisition costs are taken out of the picture, etoricoxib is still economically more favorable than celecoxib, diclofenac, or naproxen (see Table 6).

For the current economic evaluation, a comprehensive decision modeling approach was used. With this approach, an indirect comparison of efficacy and safety estimates were integrated with cost-effectiveness analysis in a single framework [18]. The advantage of this approach is that no assumptions were made regarding the uncertainty distributions used for sensitivity analysis; the Bayesian posterior uncertainty distributions of the treatment effect and GI and CV events as obtained from the MTC were directly propagated through the Markov model. The most important factor in the cost-effectiveness analysis was the probability of discontinuation as estimated with the MTC (see Figure 3) 

The etoricoxib GI safety data as used in the analysis were obtained from clinical trials in OA, RA, AS, and chronic low back pain patients [11]; the celecoxib GI safety data were obtained from the CLASS study [14]. These data were assumed applicable for AS. It could be argued that AS patients are likely younger than the average patient in the GI meta-analysis and, therefore, have a lower GI risk. However, AS patients often receive higher nsNSAID doses than patients with other arthritic conditions, thereby increasing their risk for GI events. Risk estimates of CV events were based on the MEDAL programme comparing etoricoxib with diclofenac among OA and RA patients and a meta-analysis of 12 phase II-IV clinical trials comparing etoricoxib with naproxen, among OA, RA, AS, and chronic low back pain patients [15, 30]. As AS patients are on average younger than patients in the MEDAL programme and the meta-analysis, it can be argued that the risk for CV events might have been overestimated in the model. 

In the model, the risk for a second CV event was set to be the same as before the CV event, assuming that the increased CV risk due to the history of a CV event was counterbalanced by adding aspirin to the NSAID. However, the history of a CV event might have a bigger impact than the protective effect by aspirin, which would imply an underestimation of the risk of CV events in the model, and, therefore, an underestimation of the costs due to CV events. The effect on the difference in costs, however, would be limited because the underestimation applies to both treatment initiated with etoricoxib and nsNSAIDs. 

Costs associated with severity of AS (i.e., GP visits, specialist visits, paramedical visits, hospitalization, technical examinations, adaptations and aids) were not included in the analysis. For the UK, Botteman et al. showed that each incremental change in one unit of BASDAI (0–100 scale) was estimated to be associated with a direct medical cost increase according to Cost = *£*708.45 + *£*75.00*BASDAI [51]. For the Norwegian situation, no such information was available. As a result, the cost savings of treatment of AS have probably been underestimated, especially for etoricoxib.

This evaluation was performed for the Norwegian local situation. In general, it is difficult to “transfer” cost-effectiveness estimates obtained for one country to another, due to differences in treatment practices, resource use, and unit cost data, among other. Although the cost-effectiveness findings in this study were primarily driven by differences in efficacy of the compared interventions, the cost-effectiveness of the different NSAIDs for the management of ankylosing spondylitis in other countries needs to be confirmed with country-specific analysis.

In conclusion, given the underlying assumptions and current evidence available, this economic evaluation demonstrated that etoricoxib is a cost-saving and QALY gaining therapy for AS in Norway from a health care perspective.

##  Funding

This study was funded by Merck & Co., Inc.

##  Disclosure Statement

J. P. Jansen is an employee of Mapi Values. Mapi Values received consultancy fees from Merck & Co. Inc. related to this paper. S. Taylor is an employee of Merck & Co. Inc.

## Figures and Tables

**Table 1:** Transitions between different health states of Markov model due to events and lack of efficacy.

	To						
From	Initial NSAID	Initial NSAID with PPI	Alternative nsNSAID	Alternative nsNSAID with PPI	Alternative nsNSAID with PPI & aspirin	Anti-TNF*α*	Discontinued Anti-TNF*α*
Initial NSAID	(i) No events (ii) Events w/o switch	(i) Suspected PUB (ii) Minor upper GI symptoms	(i) Edema, hypertension, hepatic, CHF, renal; all with switching treatment (ii) Lack of efficacy	Upper GI PUB	CV event	NA	NA

Initial NSAID with PPI	NA	(i) Suspected PUB (ii) Minor upper GI symptoms (iii) No events (iv) Events w/o switch	NA	(i) Upper GI (ii) PUB Edema, hypertension, hepatic, CHF, renal; all with switching tx (iii) Lack of efficacy	CV event	NA	NA

Alternative nsNSAID	NA	NA	(i) No events (ii) Events w/o switch	(i) Suspected PUB (ii) Minor upper GI symptoms	CV event	(i) Upper GI PUB (ii) Edema, hypertension, hepatic, CHF, renal; all with switching treatment (iii) Lack of efficacy	NA

Alternative nsNSAID with PPI	NA	NA	NA	(i) Suspected PUB (ii) Minor upper GI symptoms (iii) No events (iv) Events w/o switch	CV event	(i) Upper GI PUB (ii) Edema, hypertension, hepatic, CHF, renal; all with switching treatment (iii) Lack of efficacy	NA

Alternative nsNSAID with PPI & aspirin	NA	NA	NA	NA	Suspected PUB (i) Minor upper GI symptoms (ii) No events (iii) Events w/o switch	(i) Upper GI PUB (ii) CV event (iii) Edema, hypertension, hepatic, CHF, renal; all with switching treatment (iv) Lack of efficacy	NA

Anti-TNF*α*	NA	NA	NA	NA	NA	Other	Lack of efficacy

Discontinued anti-TNF*α*	NA	NA	NA	NA	NA	NA	All

**Table 2:** Individual studies and results included for mixed treatment comparison of BASFI, BASDAI and discontinuation due to lack of efficacy.

	Placebo		Celecoxib				Naproxen*		Etoricoxib		Diclofenac*	
			200 mg		400 mg		1000 mg		90 mg		150 mg	
	Mean	(SE)	Mean	(SE)	Mean	(SE)	Mean	(SE)	Mean	(SE)	Mean	(SE)
BASFI												

Barkhuizen et al. [26]	2.00	(3.00)	−9.00	(0.50)	−11.00	(1.00)	−16.00	(2.00)				
Van der Heijde et al. [16]	−4.00	(1.90)					−14.60	(1.80)	−19.40	(1.80)		
Dougados et al. [27]	1.30	(2.03)	−11.90	(2.46)								
Sieper et al. [17]			−8.00	(1.62)	−9.00	(1.23)					−9.00	(1.45)

BASDAI												

Van der Heijde et al. [16]	−6.40	(1.90)					−23.6	(1.80)	−28.60	(1.80)		
Sieper et al. [17]			−9.90	(1.71)	−13.20	(1.40)					−14.80	(1.41)

Discontinuation for lack of efficacy and (sample size)												

	*r*	*n*	*r*	*n*	*r*	*n*	*r*	*n*	*r*	*n*	*r*	*n*

Barkhuizen et al. [26]	59	156	25	137	23	161	17	157				
Van der Heijde et al. [16]	44	93					20	97	8	100		
Dougados et al. [27]	31	76	18	80								

*For mixed treatment comparison of BASDAI, the results of naproxen and diclofenac were considered as the group nsNSAID.

**Table 3:** Parameters (and distributions) for cost-effectiveness evaluation.

Parameter	Value	Uncertainty range/95% credible interval	Assumed uncertainty distribution	Source
*Efficacy*				
Change from baseline BASFI				
Etoricoxib (90 mg)	−17.87	−22.16; −13.64	No distribution assumed; posterior distributions directly obtained from mixed treatment comparison of extracted data and simultaneously forwarded into Markov model. For MTC, noninformative prior distributions were used.	Barkhuizen et al. [26]; Van der Heijde et al. [16] Dougados et al. [27]; Sieper et al. [17]
Celecoxib (200 mg)	−10.12	−12.34; −7.932		
Celecoxib (400 mg)	−11.8	−14.52; −9.10		
Diclofenac	−11.51	−15.68; −7.34		
Naproxen	−14.82	−17.69; −11.98		
Change from baseline BASDAI				
Etoricoxib (90 mg)	−28.53	−32.06; −25.05		Van der Heijde et al. [16]; Sieper et al. [17]
Celecoxib (200 mg)	−18.47	−24.12; −12.9		
Celecoxib (400 mg)	−21.77	−26.95; −16.51		
Diclofenac/naproxen	−23.46	−26.96; −19.96		
Probability of discontinuation				
Etoricoxib (90 mg)	0.063	0.027; 0.117		
Celecoxib (200 mg)	0.225	0.165; 0.292		Barkhuizen et al. [26]
Celecoxib (400 mg)	0.177	0.113; 0.255		Van der Heijde et al. [16]
Diclofenac/naproxen	0.149	0.105; 0.202		Dougados et al. [27]
BASFI without treatment	45	40; 50	Uniform (40, 50)	Based on baseline characteristics of trials included in MTC (see Table 1)
BASDAI without treatment	45	40; 50	Uniform (40, 50)	
Disease progression measured using annual changes in BASFI	0.5	0; 0.10	Uniform (0, 0.10)	Kobelt et al. [6]; Ara et al. [20]
BASFI with anti-TNF*α*	23	20; 26	Uniform (20, 26)	Ara et al. [20]
BASDAI with anti-TNF*α*	19	18; 20	Uniform (18, 20)	
BASFI when stopped with anti-TNF*α*	55	50; 60	Uniform (50, 60)	
BASDAI when stopped with anti-TNF*α*	52	47; 57	Uniform (47, 57)	
Annual probability of discontinuation from anti-TNF*α* treatment	0.10	0.05; 0.15	Beta (13.2, 118.8)	Ara et al. [20]

*Safety and treatment (annual probabilities)*				
PUBs				
Etoricoxib	0.0111	0.0074; 0.0159	No distribution assumed; posterior distribution directly obtained from indirect comparison analysis of extracted data and simultaneously forwarded into Markov model. For indirect comparison of safety, noninformative prior distributions were used.	Ramey et al. [11]; Silverstein et al. [14]
Celecoxib	0.0134	0.0075; 0.0221		
Diclofenac/naproxen	0.0270	0.0216; 0.0334		
Suspected PUBs				
Etoricoxib	0.0016	0.0000; 0.0061	No distribution assumed; posterior distribution directly obtained from analysis of extracted data and simultaneously forwarded into Markov model. For indirect comparison of safety, noninformative prior distributions were used.	Ramey et al. [11]
Celecoxib	0.0016	0.0000; 0.0061		
Diclofenac/naproxen	0.0030	0.0000; 0.0115		
Minor GI events				MEDAL study [15]
Etoricoxib/celecoxib	0.0463	0.0420; 0.0506		
Diclofenac/naproxen	0.0704	0.0650; 0.0759		

PUB risk reduction with PPI	0.40	—	—	Moore et al. [29]
Dying from PUB	0.036	—	—	Ramey et al. [11]; Laine et al. [28]
Hospitalization given PUB	0.21	0.056; 0.358	Uniform (0.056, 0.358)	Bloom et al. [39]; Maetzel et al. [40]; Knill-Jones et al. [41]; Singh and Ramey, [42]; de Pouvourville [43]; Jönsson and Haglund, [44]; Gabriel and Matteson, [45], Smalley et al. [46]; Edelson et al. [47]

Surgery given hospitalization	0.25	0.12; 0.39	Uniform (0.12, 0.39)	Maetzel et al. [40]; Knill-Jones et al. [41]; Jönsson and Haglund, [44]; Gabriel et al. [48]; Johnson et al. [49]; Kong et al. [50]
Inpatient tx given suspected PUB	0.25	0.18; 0.32	Beta (36.5, 109.5)	Maetzel et al. [40]
Treatment given minor GI	1	—	—	Assumption
Thrombotic CV event rate			No distribution assumed; posterior distribution directly obtained from indirect comparison analysis of extracted data and simultaneously forwarded into Markov model. For indirect comparison of safety, noninformative prior distributions were used.	
Etoricoxib	0.0124	0.0111; 0.01381		Cannon et al. [15]; Curtis et al. [30]
Celecoxib	0.0124	0.0111; 0.01381		
Diclofenac	0.0131	0.0117; 0.01454		
Naproxen	0.0077	0.0039; 0.01381		
Death from thrombotic CV event				Cannon et al. [15]
Etoricoxib/celecoxib	0.13	—	—	
Diclofenac/naproxen	0.128	—	—	
Edema				MEDAL [15]
Etoricoxib/celecoxib	0.0106	0.0086; 0.0127	Beta (101.4, 9459.7)	
Diclofenac/naproxen	0.0070	0.0054; 0.0088	Beta (64.9, 9165.4)	
Hypertension				
Etoricoxib/celecoxib	0.0229	0.0200; 0.0260	Beta (218.9, 9342.0)	
Diclofenac/naproxen	0.0153	0.0129; 0.0179	Beta (141.2, 9088.8)	
Coronary heart failure				
Etoricoxib/celecoxib	0.0044	0.0032; 0.0058	Beta (42.1, 9518.9)	
Diclofenac/naproxen	0.0026	0.0017; 0.0037	Beta (24.7, 9490.5)	
Hepatic events				
Etoricoxib/celecoxib	0.0036	0.0025; 0.00489	Beta (34.4, 9526.6)	
Diclofenac/naproxen	0.0218	0.0189; 0.0249	Beta (201.2, 9028.8)	
Renal events				
Etoricoxib/Celecoxib	0.0114	0.0094; 0.0136	Beta (109.0, 9452.0)	
Diclofenac/Naproxen	0.0100	0.0081; 0.0120	Beta (92.3, 9137.7)	

*Utility*				
Relation between EQ-5d and BASFI and BASDAI				Ara et al. [20]
Constant	0.924	0.890; 0.957	Normal (0.924, 0.017^2^)
BASFI	−0.004	−0.0057; −0.0029	Normal (−0.004, 0.0007^2^)
BASDAI	−0.004	−0.0056; −0.0024	Normal (−0.004, 0.0008^2^)

*Disutility due to adverse events (adjusted for duration)*				
Surgery for PUB	0.080	0.069; 0.092	Beta distributions	Moore et al. [29]
Inpatient treatment for PUB	0.062	0.052; 0.072		
Outpatient treatment for PUB	0.051	0.042; 0.060		
Inpatient investigation for suspected PUB	0.062	0.052; 0.072		
Outpatient investigation for suspected PUB	0.025	0.021; 0.030		
Minor GI symptoms requiring treatment	0.015	0.012; 0.019		
Minor GI symptoms not requiring treatment	0.00004	0.00000; 0.00032		
Thrombotic CV event	0.294	0.256; 0.331		Moore et al. [29]
Edema	0.020	0.016; 0.024		Revicki [33]
Hypertension	0.001	0.000; 0.002		Stason and Weinstein, [34]
Hepatic	0.055	0.040; 0.072		Nichol et al. [35]
CHF	0.002	0.001; 0.002		Wong et al, [36]
Renal	0.020	0.016; 0.024		Revicki [33]

*Costs of events (NOK)*				
Surgery for PUB	22,904	18,900; 27,300		
Inpatient treatment for PUB	22,904	18,900; 27,300		
Outpatient treatment for PUB	2,231	2,038; 2,437	Gamma distributions	Resource use from Jansen et al. [19]; drug acquisition costs from NoMA (September 2007); GP costs from Norwegian Medical Association; DRG prices from ISF 2007 [37];
Inpatient investigation for suspected PUB	22,295	18,240; 26,700		
Outpatient investigation for suspected PUB	1,297	1,157; 1,445		
Minor GI symptoms requiring treatment	568	507; 636		
Thrombotic CV event	95,555	—	—	NoMA (September 2007); ISF 2007 [37];
CHF	45,958	—	—	NoMA (September 2007); ISF 2007 [37];

*Annual drug costs (NOK)*				
Etoricoxib (90 mg)	4,654	—	—	NoMA (September 2007)
Celecoxib (200 mg)	3,318	—	—	
Celecoxib (400 mg)	6,636	—	—	
Diclofenac (150 mg)	1,588	—	—	
Naproxen (1000 mg)	1,380	—	—	
PPI (omeprazole)	3,050	—	—	
Aspirin (75 mg)	383	—	—	
Anti-TNF*α* tx cost	143,322	—	—	NoMA (September 2007)

**Table 4:** Estimated effects and costs by treatment (base-case scenario).

	Etoricoxib (90 mg)			Celecoxib (200 & 400 mg)			Diclofenac (150 mg)			Naproxen (1000 mg)		
	Estimate	95% CrI		Estimate	95% CrI		Estimate	95% CrI		Estimate	95% CrI	
*Life years*												
1 yrs	1	1	1	1	1	1	1	1	1	1	1	1
5 yrs	4.59	4.59	4.60	4.59	4.59	4.60	4.59	4.59	4.59	4.59	4.59	4.60
30 yrs	16.72	16.69	16.76	16.76	16.73	16.79	16.73	16.69	16.76	16.76	16.71	16.80
*QALYs*												
1 yrs	0.74	0.66	0.80	0.67	0.59	0.75	0.69	0.61	0.76	0.70	0.62	0.77
5 yrs	3.34	3.02	3.64	3.14	2.78	3.48	3.17	2.82	3.50	3.22	2.88	3.54
30 yrs	11.16	9.85	12.42	10.66	9.24	12.04	10.71	9.30	12.08	10.80	9.41	12.15

*P* (best)^2^	98.90%	at 1 year		0.00%	at 1 year		0.13%	at 1 year		0.98%	at 1 year	
	98.94%	at 5 years		0.00%	at 5 years		0.11%	at 5 years		0.95%	at 5 years	
	99.96%	at 30 years		0.00%	at 30 years		0.00%	at 30 years		0.04%	at 30 years	

*Treatment cost (NOK)*												
1 yrs	4,654	4,654	4,654	4,977	4,977	4,977	1,588	1,588	1,588	1,380	1,380	1,380
5 yrs	50,020	41,400	62,060	74,940	62,790	89,160	60,250	44,380	80,180	59,030	43,100	79,000
30 yrs	628,200	463,300	823,600	740,500	544,400	971,100	710,400	517,500	937,800	708,900	516,200	936,000

*GI event costs (NOK)*												
1 yrs	109	69	158	124	65	214	236	144	341	236	144	341
5 yrs	554	360	784	648	391	977	939	576	1,356	943	579	1,361
30 yrs	1,159	743	1,644	1,069	654	1,579	1,453	873	2,148	1,469	881	2,173

*Thrombotic CV event costs (NOK)*												
1 yrs	1,148	1,027	1,278	1,148	1,027	1,278	1,208	1,082	1,345	714	360	1,271
5 yrs	5,065	4,617	5,542	4,871	4,476	5,289	5,081	4,535	5,672	3,654	2,590	5,309
30 yrs	10,060	8,452	11,650	7,696	6,712	8,756	8,293	6,852	9,920	6,541	4,888	8,833

*Other AE costs (NOK)*												
1 yrs	202	146	268	202	146	268	120	77	171	120	77	171
5 yrs	814	612	1,048	723	555	918	502	323	718	504	324	720
30 yrs	1,507	1,098	1,991	1,048	795	1,342	820	513	1,201	828	517	1,214

*Total costs (NOK)*												
1 yrs	6,115	5,974	6,267	6,452	6,300	6,618	3,152	2,981	3,332	2,449	2,073	3,015
5 yrs	56,450	47,920	68,450	81,190	69,120	95,230	66,780	51,010	86,440	64,130	48,120	84,220
30 yrs	640,900	476,900	835,600	750,300	554,500	980,800	721,000	528,800	947,700	717,800	526,000	944,700

*P* (best)^2^	0.00%	at 1 year		0.00%	at 1 year		1.15%	at 1 year		98.85%	at 1 year	
	85.26%	at 5 years		0.00%	at 5 years		0.18%	at 5 years		14.57%	at 5 years	
	98.92%	at 30 years		0.01%	at 30 years		0.03%	at 30 years		1.04%	at 30 years	

^1^All results are discounted, 4.0% for effects and costs.

^2^Probability that a certain intervention provides best outcomes (i.e., greatest QALYs, lowest costs).

**Table 5:** Cost-effectiveness of etoricoxib relative to other interventions (base-case scenario).

	Incremental costs in NOK			Incremental QALYs			Incremental cost-effectiveness ratio		
	Estimate	95% CrI		Estimate	95% CrI		Estimate	95% CrI	
*1 year*									
Etoricoxib (90 mg) versus celecoxib (200 & 400 mg)	−337	−411	−280	0.06	0.03	0.10	Dominant	Dominant	Dominant
Etoricoxib (90 mg) versus diclofenac	2,964	2,753	3,173	0.05	0.02	0.09	59,221	33,180	184,500
Etoricoxib (90 mg) versus naproxen	3,666	3,109	4,046	0.03	0.01	0.07	107,256	51,320	494,300

*5 years*									
Etoricoxib (90 mg) versus celecoxib (200 & 400 mg)	−24,730	−37,730	−11,720	0.20	0.08	0.33	Dominant	Dominant	Dominant
Etoricoxib (90 mg) versus diclofenac	−10,320	−26,070	2,840	0.17	0.05	0.30	Dominant	Dominant	Dominant
Etoricoxib (90 mg) versus naproxen	−7,682	−23,540	5,729	0.12	0.02	0.23	Dominant	Dominant	Dominant

*30 years*									
Etoricoxib (90 mg) versus celecoxib (200 & 400 mg)	−109,400	−198,700	−38,640	0.51	0.25	0.84	Dominant	Dominant	Dominant
Etoricoxib (90 mg) versus diclofenac	−80,060	−164,800	−13,280	0.45	0.20	0.76	Dominant	Dominant	Dominant
Etoricoxib (90 mg) versus naproxen	−76,850	−162,100	−9,622	0.36	0.13	0.66	Dominant	Dominant	Dominant

**Table 6:** Cost-effectiveness of etoricoxib relative to other interventions when anti-TNF*α* costs are set to zero.

	Incremental costs in NOK			Incremental QALYs			ICER		
	Estimate	95% CrI		Estimate	95% CrI		Estimate	95% CrI	
*1 year*									
Etoricoxib (90 mg) versus celecoxib (200 & 400 mg)	−337	−410	−280	0.06	0.03	0.10	Dominant	Dominant	Dominant
Etoricoxib (90 mg) versus diclofenac	2,965	2,756	3,173	0.05	0.02	0.09	59,288	33,190	188,200
Etoricoxib (90 mg) versus naproxen	3,663	3,099	4,043	0.03	0.01	0.07	107,074	50,970	488,500
*5 years*									
Etoricoxib (90 mg) versus celecoxib (200 & 400 mg)	2,194	426	3,745	0.20	0.08	0.33	10,926	2083	30820
Etoricoxib (90 mg) versus diclofenac	10,560	9,058	11,880	0.17	0.05	0.30	62,411	34650	198400
Etoricoxib (90 mg) versus naproxen	12,370	10,330	14,000	0.12	0.02	0.23	103,083	49220	465100
*30 years*									
Etoricoxib (90 mg) versus celecoxib (200 & 400 mg)	15,740	5,398	25,490	0.51	0.25	0.84	31,009	14330	59810
Etoricoxib (90 mg) versus diclofenac	23,910	14,520	32,990	0.45	0.20	0.76	53,181	34020	103800
Etoricoxib (90 mg) versus naproxen	25,660	16,150	34,810	0.36	0.13	0.66	70,825	42830	170900

## References

[1] Gossec L, van der Heijde D, Melian A (2005). Efficacy of cyclo-oxygenase-2 inhibition by etoricoxib and naproxen on the axial manifestations of ankylosing spondylitis in the presence of peripheral arthritis. *Annals of the Rheumatic Diseases*.

[2] Saraux A, Guillemin F, Guggenbuhl P (2005). Prevalence of spondyloarthropathies in France: 2001. *Annals of the Rheumatic Diseases*.

[3] Braun J, Bollow M, Remlinger G (1998). Prevalence of spondylarthropathies in HLA-B27 positive and negative blood donors. *Arthritis and Rheumatism*.

[4] Akkoc N, Khan MA, Braun J, Listing J, Sieper J (2005). Overestimation of the prevalence of ankylosing spondylitis in the Berlin study: comment on the article by Braun et al. *Arthritis and Rheumatism*.

[5] Boonen A, van der Heijde D, Landewé R (2003). Direct costs of ankylosing spondylitis and its determinants: an analysis among three European countries. *Annals of the Rheumatic Diseases*.

[6] Kobelt G, Andlin-Sobocki P, Brophy S, Jönsson L, Calin A, Braun J (2004). The burden of ankylosing spondylitis and the cost-effectiveness of treatment with infliximab (Remicade ®). *Rheumatology*.

[7] Sieper J, Braun J (2001). New treatment options in ankylosing spondylitis: a role for anti-TNF*α* therapy. *Annals of the Rheumatic Diseases*.

[8] Braun J, Pham T, Sieper J (2003). International ASAS consensus statement for the use of anti-tumour necrosis factor agents in patients with ankylosing spondylitis. *Annals of the Rheumatic Diseases*.

[9] Zochling J, van der Heijde D, Dougados M, Braun J (2006). Current evidence for the management of ankylosing spondylitis: a systematic literature review for the ASAS/EULAR management recommendations in ankylosing spondylitis. *Annals of the Rheumatic Diseases*.

[10] Keat A, Barkham N, Bhalla A (2005). BSR guidelines for prescribing TNF-*α* blockers in adults with ankylosing spondylitis. Report of a working party of the British Society for Rheumatology. *Rheumatology*.

[11] Ramey DR, Watson DJ, Yu C, Bolognese JA, Curtis SP, Reicin AS (2005). The incidence of upper gastrointestinal adverse events in clinical trials of etoricoxib vs. non-selective NSAIDs: an updated combined analysis. *Current Medical Research and Opinion*.

[12] Hunt RH, Harper S, Callegari P (2003). Complementary studies of the gastrointestinal safety of the cyclo-oxygenase-2-selective inhibitor etoricoxib. *Alimentary Pharmacology and Therapeutics*.

[13] Hunt RH, Harper S, Watson DJ (2003). The gastrointestinal safety of the COX-2 selective inhibitor etoricoxib assessed by both endoscopy and analysis of upper gastrointestinal events. *American Journal of Gastroenterology*.

[14] Silverstein FE, Faich G, Goldstein JL (2000). Gastrointestinal toxicity with Celecoxib vs nonsteroidal anti-inflammatory drugs for osteoarthritis and reumatoid arthritis: the CLASS study: a randomized controlled trial. *Journal of the American Medical Association*.

[15] Cannon CP, Curtis SP, FitzGerald GA (2006). Cardiovascular outcomes with etoricoxib and diclofenac in patients with osteoarthritis and rheumatoid arthritis in the Multinational Etoricoxib and Diclofenac Arthritis Long-term (MEDAL) programme: a randomised comparison. *The Lancet*.

[16] van der Heijde D, Baraf HSB, Ramos-Remus C (2005). Evaluation of the efficacy of etoricoxib in ankylosing spondylitis: results of a fifty-two-week, randomized, controlled study. *Arthritis and Rheumatism*.

[17] Sieper J, Klopsch T, Richter M (2008). Comparison of two different dosages of celecoxib with diclofenac for the treatment of active ankylosing spondylitis: results of a 12-week randomised, double-blind, controlled study. *Annals of the Rheumatic Diseases*.

[18] Cooper NJ, Sutton AJ, Abrams KR (2002). Decision analytical economic modelling within a Bayesian framework: application to prophylactic antibiotics use for caesarean section. *Statistical Methods in Medical Research*.

[19] Jansen JP, Gaugris S, Choy EH, Ostor A, Nash JT, Stam W (2010). Cost-effectiveness evaluation of etoricoxib versus celecoxib and non-selective NSAIDs in the treatment of ankylosing spondylitis. *PharmacoEconomics*.

[20] Ara RM, Reynolds AV, Conway P (2007). The cost-effectiveness of etanercept in patients with severe ankylosing spondylitis in the UK. *Rheumatology*.

[21] Lu G, Ades AE (2004). Combination of direct and indirect evidence in mixed treatment comparisons. *Statistics in Medicine*.

[22] Jansen JP, Crawford B, Bergman G, Stam W (2008). Bayesian meta-analysis of multiple treatment comparisons: an introduction to mixed treatment comparisons. *Value in Health*.

[23] Boonen A, van der Heijde D, Severens JL (2006). Markov model into the cost-utility over five years of etanercept and infliximab compared with usual care in patients with active ankylosing spondylitis. *Annals of the Rheumatic Diseases*.

[24] Taylor AL, Balakrishnan C, Calin A (1998). Reference centile charts for measures of disease activity, functional impairment, and metrology in ankylosing spondylitis. *Arthritis and Rheumatism*.

[25] Kristensen LE, Saxne T, Geborek P (2006). The LUNDEX, a new index of drug efficacy in clinical practice: results of a five-year observational study of treatment with infliximab and etanercept among rheumatoid arthritis patients in Southern Sweden. *Arthritis and Rheumatism*.

[26] Barkhuizen A, Steinfeld S, Robbins J, West C, Coombs J, Zwillich S (2006). Celecoxib is efficacious and well tolerated in treating signs and symptoms of ankylosing spondylitis. *Journal of Rheumatology*.

[27] Dougados M, Béhier JM, Jolchine I (2001). Efficacy of celecoxib, a cyclooxygenase 2-specific inhibitor, in the treatment of ankylosing spondylitis: a six-week controlled study with comparison against placebo and against a conventional nonsteroidal antiinflammatory drug. *Arthritis and Rheumatism*.

[28] Laine L, Curtis SP, Cryer B, Kaur A, Cannon CP (2007). Assessment of upper gastrointestinal safety of etoricoxib and diclofenac in patients with osteoarthritis and rheumatoid arthritis in the Multinational Etoricoxib and Diclofenac Arthritis Long-term (MEDAL) programme: a randomised comparison. *The Lancet*.

[29] Moore A, Phillips C, Hunsche E, Pellissier J, Crespi S (2004). Economic evaluation of etoricoxib versus non-selective NSAIDs in the treatment of osteoarthritis and rheumatoid arthritis patients in the UK. *PharmacoEconomics*.

[30] Curtis SP, Mukhopadhyay  S, Ramey D (2003). Cardiovascular safety summary associated with the etoricoxib development program. *Arthritis & Rheumatism*.

[31] Tramèr MR, Moore RA, Reynolds DJM, McQuay HJ (2000). Quantitative estimation of rare adverse events which follow a biological progression: a new model applied to chronic NSAID use. *Pain*.

[32] Life Tables for WHO Member States http://www.who.int/healthinfo/statistics/mortality_life_tables/en/.

[33] Revicki DA (1992). Relationship between health utility and psychometric health status measures. *Medical Care*.

[34] Stason WB, Weinstein MC (1977). Allocation of resources to manage hypertension. *The New England Journal of Medicine*.

[35] Nichol G, Kaul P, Huszti E, Bridges JFP (2004). Cost-effectiveness of cardiac resynchronization therapy in patients with symptomatic heart failure. *Annals of Internal Medicine*.

[36] Wong JB, Koff RS, Tine F, Pauker SG (1995). Cost-effectiveness of interferon-*α*2b treatment for hepatitis B e antigen- positive chronic hepatitis B. *Annals of Internal Medicine*.

[37] ISF 2007 Ministry of Health and Care Services. http://www.helsedirektoratet.no/vp/multimedia/archive/00014/IS-1439_14578a.pdf.

[38] Sonnenberg FA, Beck JR (1993). Markov models in medical decision making: a practical guide. *Medical Decision Making*.

[39] Bloom BS (1988). Direct medical costs of disease and gastrointestinal side effects during treatment for arthritis. *The American Journal of Medicine*.

[40] Maetzel A, Ferraz MB, Bombardier C (1998). The cost-effectiveness of misoprostol in preventing serious gastrointestinal events associated with the use of nonsteroidal antiinflammatory drugs. *Arthritis and Rheumatism*.

[41] Knill-Jones R, Drummond M, Kohli H, Davies L (1990). Economic evaluation of gastric ulcer prophylaxis in patients with arthritis receiving non-steroidal anti-inflammatory drugs. *Postgraduate Medical Journal*.

[42] Singh G, Ramey DR (1998). NSAID induced gastrointestinal complications: the ARAMIS perspective—1997. *Journal of Rheumatology*.

[43] de Pouvourville G (1992). The economic consequences of NSAID-induced gastropathy: the French context. *Scandinavian Journal of Rheumatology, Supplement*.

[44] Jönsson B, Haglund U (1992). Cost-effectiveness of misoprostol in Sweden. *International Journal of Technology Assessment in Health Care*.

[45] Gabriel SE, Matteson EL (1995). Economic and quality-of-life impact of NSAIDs in rheumatoid arthritis. A conceptual framework and selected literature review. *PharmacoEconomics*.

[46] Smalley WE, Griffin MR, Fought RL, Ray WA (1996). Excess costs from gastrointestinal disease associated with nonsteroidal anti-inflammatory drugs. *Journal of General Internal Medicine*.

[47] Edelson JT, Tosteson ANA, Sax P (1990). Cost-effectiveness of misoprostol for prophylaxis against nonsteroidal anti-inflammatory drug-induced gastrointestinal tract bleeding. *Journal of the American Medical Association*.

[48] Gabriel SE, Campion ME, O’Fallon WM (1993). Patient preferences for nonsteroidal antiinflammatory drug related gastrointestinal complications and their prophylaxis. *Journal of Rheumatology*.

[49] Johnson RE, Hornbrook MC, Hooker RS, Woodson GT, Shneidman R (1997). Analysis of the costs of NSAID-associated gastropathy. Experience in a US health maintenance organisation. *PharmacoEconomics*.

[50] Kong SX, Hatoum HT, Zhao SZ, Agrawal NM, Geis SG (1998). Prevalence and cost of hospitalization for gastrointestinal complications related to peptic ulcers with bleeding or perforation: comparison of two national databases. *American Journal of Managed Care*.

[51] Botteman MF, Hay JW, Luo MP, Curry AS, Wong RL, van Hout BA (2007). Cost effectiveness of adalimumab for the treatment of ankylosing spondylitis in the United Kingdom. *Rheumatology*.

